# Cirrhosis, Liver Transplantation and HIV Infection Are Risk Factors Associated with Hepatitis E Virus Infection

**DOI:** 10.1371/journal.pone.0103028

**Published:** 2014-07-28

**Authors:** Mar Riveiro-Barciela, María Buti, María Homs, Isabel Campos-Varela, Carmen Cantarell, Manuel Crespo, Lluís Castells, David Tabernero, Josep Quer, Rafael Esteban, Francisco Rodriguez-Frías

**Affiliations:** 1 Liver Unit, Internal Medicine Department, Hospital Universitario Vall d'Hebron, Barcelona, Spain; 2 Centro de Investigación Biomédica en Red de Enfermedades Hepáticas y Digestivas (CIBERehd), Hospital Universitario Vall d'Hebron, Barcelona, Spain; 3 Liver Pathology Unit/Virology Unit Biochemistry and Microbiology Departments, Hospital Universitario Vall d'Hebron, Barcelona, Spain; 4 Nephrology Department, Hospital Universitario Vall d'Hebron, Barcelona, Spain; 5 Infectious Diseases Department, Hospital Universitario Vall d'Hebron, Barcelona, Spain; Virginia Polytechnic Institute and State University, United States of America

## Abstract

**Background:**

Acute and chronic hepatitis E have been associated with high mortality and development of cirrhosis, particularly in solid-organ recipients and patients infected by human immunodeficiency virus. However, data regarding the epidemiology of hepatitis E in special populations is still limited.

**Aims:**

Investigate seroprevalence and possible factors associated with HEV infection in a large cohort of immunosuppressed patients.

**Methods:**

Cross-sectional study testing IgG anti-HEV in serum samples from 1373 consecutive individuals: 332 liver-transplant, 296 kidney-transplant, 6 dual organ recipients, 301 non-transplanted patients with chronic liver disease, 238 HIV-infected patients and 200 healthy controls.

**Results:**

IgG anti-HEV was detected in 3.5% controls, 3.7% kidney recipients, 7.4% liver transplant without cirrhosis and 32.1% patients who developed post-transplant cirrhosis (*p*<0.01). In patients with chronic liver disease, IgG anti-HEV was also statistically higher in those with liver cirrhosis (2% vs 17.5%, *p*<0.01). HIV-infected patients showed an IgG anti-HEV rate of 9.2%, higher than those patients without HIV infection (*p*<0.03). Multivariate analysis showed that the factors independently associated with anti-HEV detection were liver cirrhosis, liver transplantation and HIV infection (OR: 7.6, 3.1 and 2.4). HCV infection was a protective factor for HEV infection (OR: 0.4).

**Conclusions:**

HEV seroprevalence was high in liver transplant recipients, particularly those with liver cirrhosis. The difference in anti-HEV prevalence between Liver and Kidney transplanted cases suggests an association with advanced liver disease. Further research is needed to ascertain whether cirrhosis is a predisposing factor for HEV infection or whether HEV infection may play a role in the pathogeneses of cirrhosis.

## Introduction

Hepatitis E represents a major public health problem especially in developing countries, where the mortality rate is 1–15% and up to 30% in pregnant women [1]. In industrialized countries Hepatitis E virus (HEV) infection was first described as sporadic acute hepatitis E infections detected in travelers from endemic areas. More recently, an increasing number of autochthonous hepatitis E cases have been reported in developed countries. Most of these are due to HEV genotype 3, and have been related to a high mortality rate, mainly in those patients developing acute-on-chronic liver failure [2], [3]. In 2008, the first cases of chronic infection E were described [4], that can lead to the development of hepatic fibrosis and even cirrhosis in immunosuppressed patients such as human immunodeficiency virus (HIV)-infected and solid-organ-transplant recipients.

The epidemiology of HEV is more complex than was initially appreciated, and many features remain unexplained, though zoonosis seems to be the main way of transmission. Some cases of acute transfusion-transmitted hepatitis E infections [5], [6], [7], [8], [9], [10], [11], have led to an increasing number of publications reporting the prevalence of serum Ig G antibodies to HEV (anti-HEV) in blood donors in western countries. These rates oscillate betweeen 4.7% in Scotland [12] to up to 52% in adults from from Midi-Pyrénées [13]. However, regarding industrialized countries, it should be stressed that important differences in the prevalence rates have been described and related to age, geographic region and even the anti-HEV assay used [14], [15].

There are few reports regarding HEV seroprevalence in immunocompromised individuals [3], [16], [17], [18], [19], [20], [21], [22], [23], [24], [25], [26]. Superinfection with other hepatitis viruses is associated with progression of liver diseases, since multiple hepatotropic viruses infecting a single patient may amplify liver damage. In endemic areas, similar to hepatitis A infection, it has been described a high seroprevalence of HEV and a more severe hepatitis related to HEV infection in patients with pre-existing chronic liver disease (CLD) due to hepatitis B virus (HBV) or hepatitis C virus (HCV) [27]. Moreover, in USA, a significant association between HEV seropositivity and antibodies to HCV has been also reported [28]. More recently, a study performed by Pischke *et al* revealed a high anti-HEV IgG rate in patients diagnosed with autoimmune hepatitis, suggesting that hepatitis E infection could have triggered immune events in those patients [29].

Therefore, in order to better understand the clinical impact of HEV infection in these populations we have conducted a study determining IgG anti-HEV in a cohort of solid-organ transplant recipients (liver and kidney), individuals with CLD including end-stage liver disease, HIV-infected patients and healthy controls. In addition, epidemiological, clinical and analytical factors were analyzed in order to identify potential risk factors associated with HEV seropositivity.

## Patients and Methods

Cross-sectional study carried out at the Hospital Universitario Vall d'Hebron in Barcelona.

1173 patients consecutively attended at the outpatients clinic from January 2011 to May 2011 were enrolled: **1**) 244 patients with CLD: 117 HCV, 119 HBV, 2 dual infection, 6 non-viral CLD; **2**) 101 non-transplanted patients with cirrhosis (62 HCV, 19 HBV, 1 dual infection, 19 non-viral cirrhosis); **3**) 338 liver transplant recipients (28 with post-transplantation cirrhosis; 6 kidney-liver transplantation); **4**) 238 HIV patients treated with highly active antiretroviral therapy (HAART): 118 coinfected by HCV, 9 HBV, 11 HCV and HBV (44 end-stage liver disease), 100 without hepatitis; **5**) 296 kidney transplant recipients. Two hundred serum samples from healthy individuals were studied as a control group. Data from the HIV-infected patients cohort was previously published [30], and is currently used to increase the number of immunocompromised patients and to improve the quality of the present study. The cohort of HIV patients was previously analysed [30] but not compared with other immunosuppressed patients. This comparison has been performed in the present study.

All solid-organ recipients underwent transplantation in our hospital at adult age, between November 1991 and December 2010. At the moment of inclusion, they received standard immunosuppressive regimens consisting of a calcineurin inhibitor (mainly tacrolimus), steroids and/or mycophenolate and mTOR (syrolimus or everolimus).

In the study the possible association between the characteristics of the patients: CLD, LC, immunosuppressed status, HBV or HCV serological markers, gender, and alanine aminotransferase (ALT) levels with anti-HEV positivity were analyzed by univariate and multivariate logistic regression analysis. Verbal informed consent for participation in the study was obtained and written in the medical records from all patients at the time of blood sampling, as HEV screening was considered a part of the serological study in patients suffering from liver disease. Moreover, healthy controls also signed a written consent. The Ethics Committee of Vall d'Hebron Research Institute approved this study.

## Methods

Antibodies to HEV were determined in serum samples by enzyme immunoassay (EIA) (Bioelisa HEV IgG 3.0 Biokit, Barcelona, Spain; assay based on the MP Diagnostics, Singapore, former Genelabs) according to the manufacturer's instructions. As previously reported [26], [30], [31], [32], [33], [34], this assay uses type-common recombinant HEV antigens from the structural region of the viral genome, derived from Burmese and Mexican strains. A repeatedly positive result indicates the presence of anti-HEV. Serum samples were analyzed for hepatitis B surface antigen (HBsAg), anti-HCV, and anti-HIV using commercial enzyme immunoassays: Vitros HBsAg, Vitros anti-HCV (Johnson & Johnson, Rochester, NY, USA and Enzygnost HIV Integral II (Siemens Health Care Diagnosis. Germany).

Demographic and clinical data were collected from the medical records at the time of blood sample extraction. The diagnosis of CLD was established by persistent high ALT levels concomitant to viral infection or cofactors such as alcohol intake. LC was diagnosed based on histological examination or a combination of clinical, biochemical (AST/Platelet Ratio Index >2), transient elastography >14 kPa and ultrasound imaging findings.

### Statistical Analysis

Statistical analyses were performed using the statistical software package SPSS for Windows, version 19.0 (SPSS, Chicago, IL). Continuous variables are expressed as the median and interquartile range (IQR) or mean and standard deviation, as appropriate, and were compared using the Student *t*-test or the Mann–Whitney *U*-test. Categorical variables were compared using the X^2^ test or the Fisher exact test. In order to assess the importance of some variables such as the presence of liver cirrhosis, some of the univariate comparisons were performed using data from particular groups. Variables with statistical significance or with *P*<0.10 in the univariate model were analyzed in a multivariate logistic regression model. Odds ratios (OR) and 95% CI were calculated for the independent predictive factors of Ig G HEV antibody positivity. For the multivariate logistic regression analysis performed, only patients with available data for all the variables taken into account for the analysis were included. A *P*-value <0.05 was considered statistically significant.

## Results

Anti-HEV antibodies were detected in 7 out of 200 healthy controls (3.5%), 11 out of 296 kidney-transplant recipients (3.7%), 15 out of 301 non-transplant CLD (5%), 22 out of 238 HIV-infected patients (9.2%) and 32 out of 338 liver-transplant recipients (9.5%). Table 1 summarizes the anti-HEV seroprevalence and the epidemiological baseline characteristics observed in the different groups based on the presence of liver disease.

**Table 1:** Clinical baseline characteristics and anti-HEV seroprevalence of the different groups based on the presence of liver disease.

	Chronic liver disease		Immunocompromised patients				
	No liver cirrhosis	Liver cirrhosis	HIV-infected patients		Liver transplant		Kidney transplant
			No liver disease	Liver disease	No cirrhosis	Cirrhosis	
Patients	244	57	98	140	310	28	296
Age (years)	49 (39–60)	55 (42–64)	43 (34–49.3)	45 (40–49)	59 (50–66)	59 (47–67)	54.5 (40–65)
Male sex	152 (62.3)	31 (54.4)	69 (70.4)	105 (75)	208 (67.1)	19 (67.9)	180 (60.8)
ALT level (IU/L)	35.5 (20–56)	47 (24–117.5)	25.5 (17–36)	35.5 (24–63.3)	35 (21.8–76)	49 (28–85.8)	20 (14–27)
**Anti-HEV IgG**	**5 (2%)**	**10 (17.5%)**	**8 (8.2%)**	**14 (10%)**	**23 (7.4%)**	**9 (32.1%)**	**11 (3.7%)**

ALT: alanine aminotransferase; HEV: hepatitis E virus; HIV: human immunodeficiency virus; IU/L: international units per liter.

Results are expressed as median (IQR) or n (%).

Liver transplantation (LT) was related to HBV, HCV or HBV/HCV induced-cirrhosis in 36 (10.7%), 172 (50.9%) and 7 (2.1%) cases, respectively, alcoholic-liver cirrhosis in 69 (20.4%), primary biliary cirrhosis in 18 (5.3%), hepatocellular carcinoma in 16 (4.7%), and other causes in 20 patients (5.9%). Regarding kidney transplantation (KT), the main causes of end-stage renal disease were glomerular disease in 89 (30%) of cases, polycystic kidney disease in 39 (13.2%) and diabetes mellitus in 44 (15%). Dual liver and kidney-transplantation was needed in six patients: three cases of HCV infection, a case of HBV infection and two alcohol related LC. From the remaining 296 KT recipients, 36 patients also suffered from HCV infection and 1 dual HCV/HBV infection, but without signs of LC. The median months between orthotropic liver transplantation (OLT) and the obtained serum samples was 48 (1–228).

At the moment of the study, 28 of LT recipients already presented liver cirrhosis: 23 related to HCV, 1 HBV, 3 alcoholic-related LC and one case of non-alcoholic steatohepatitis (NASH).

### Anti-HEV seroprevalence: Univariant analysis

The factors associated in the univariant analysis to a higher IgG anti-HEV rate were immunosuppression, liver disease, liver transplantation, liver cirrhosis and HIV infection. A summary of univariate analysis of factors associated with a higher IgG anti-HEV seroprevalence are summarized in table 2.

**Table 2:** Univariate analysis of factors independently associated with anti-HEV seroprevalence.

Factor	Variables	N	HEV (+)	(%)	p
**Gender**	Male	886	62	7%	0.11
	Female	487	25	5.1%	
**Median age ¥**	<51 years old	596	38	6.4%	0.3
	>51 years old	576	42	7.3%	
**Healthy controls**	Healthy individuals	200	7	3.5%	0.03
	Immunocompromised	872	65	7.5%	
**HIV infection**	HIV-infected	238	22	9.2%	0.03
	Non-HIV-infected	1135	65	5.7%	
**Solid organ transplantation**	Kidney	296	11	3.7%	<0.01
	Liver	338	32	9.5%	
**No liver disease Ж**	HIV-infected	98	8	8.2%	0.07
	Non-HIV-infected	459	18	3.9%	
**HIV infection Ж**	No liver disease	98	8	8.2%	0.4
	Liver disease	140	14	10%	
	No cirrhosis	194	12	6.2%	<0.01
	Cirrhosis	44	10	22.7%	
**OLT Ж**	Post-transplant LC	28	9	32.1%	<0.01
	No LC	310	23	7.4%	
	Non HCV	159	16	10.1%	0.43
	HCV	179	16	8.9%	
**Liver cirrhosis Ж**	HIV-infected	44	10	22.7%	0.56
	Non-HIV-infected	85	19	22.4%	
**Liver cirrhosis (HIV- infected included)Ж**	Non-OLT	101	20	19.8%	0.13
	OLT	28	9	32.1%	
**Liver disease (CLD after OLT included) Ж**	No liver cirrhosis	687	32	4.7%	<0.01
	Liver cirrhosis	129	29	22.5%	
	Non-HCV	330	30	9.1%	0.1
	HCV	486	31	6.4%	
	Non-HBV	613	44	7.2%	0.34
	HBV	203	17	8.4%	

CLD: Chronic liver disease; HBV; hepatitis B virus; HCV: hepatitis C virus; HEV: hepatitis E virus; HIV: human immunodeficiency virus; LC: liver cirrhosis; OLT: orthotopic liver transplantation.

Ж Only data from individuals having this factor were included.

Regarding HIV-infected patients, the global IgG anti-HEV rate of this group was 9.2% and statistically higher than other patients under immunosuppressant therapy such as KT recipients (*p* = 0.01). HIV-infected patients affected also by CLD presented a higher anti-HEV seroprevalence than those who were not affected (10% vs 8.2%, *p* = 0.4), a difference which reached statistical significance when the presence of liver cirrhosis was compared (22.7% vs 6.2%, *p*<0.01). Although all patients underwent HAART, some of them presented a CD4+ lymphocyte count lower than 200 cells per µL. In this group, the IgG anti-HEV rate was higher, though this difference did not reach statistical significance when it was contrasted with patients whose CD4+ count was higher than 200 cells per µL (15.4% vs 8.5%, *p* = 0.2).

With regards to liver transplant recipients, the development of post-transplant liver cirrhosis was also an independent factor associated with a higher percentage of anti-HEV. It is important to stress that all cases of OLT were transplanted at end-stage liver disease. After OLT, twenty eight cases of post-transplant LC were observed. Nine out of 28 patients presented anti-HEV positivity (32.1%), which was statistically higher than 7.4% seroprevalence in the OLT without current LC (*p*<0.01). It should be noted that two out of four patients with non-viral liver disease presented positive anti-HEV antibodies (50%). In addition, seven of the 23 (30.4%) HCV related post-LT LC cases were positive for anti-HEV. No significant differences in anti-HEV seroprevalence were observed in relation to the time elapsed after OLT.

Overall, patients with liver disease presented a higher seroprevalence of anti-HEV than those individuals without evidence of liver damage. Patients infected by HCV presented a lower seroprevalence though this difference did not reach statistical significance (6.4% vs 9.1%, *p* = 0.1). OLT was statistically associated to a higher anti-HEV seroprevalence, but KT was not, though both groups of patients underwent immunosuppressant therapy.

In order to contrast the importance of continuous variables such as ALT levels or age, the median value was used. However, no significant differences were found for age, gender, country of origin, race or ALT levels.

### Anti-HEV seroprevalence: Multivariant analysis

The main factor associated with a higher anti-HEV seroprevalence was the presence of liver cirrhosis. Patients diagnosed with LC presented anti-HEV antibodies at a rate 7 times higher (OR 7.6, 95% CI: 4.4–13.1). The other factors independently related to a higher anti-HEV seroprevalence were liver transplantation and HIV infection (Table 3). Nevertheless, HCV infection was associated with a lower HEV seroprevalence rate.

**Table 3:** Multivariate analysis of factors independently associated with anti-HEV seroprevalence.

	OR	95% CI	p
**Liver cirrhosis**	7.6	4.4–13.1	<0.001
**OLT**	3.1	1.8–5.4	<0.001
**HCV infection**	0.4	0.3–0.8	0.003
**HIV infection**	2.4	1.3–4.4	0.006

HCV: hepatitis C virus; HIV: human immunodeficiency virus; OLT: orthotopic liver transplantation.

## Discussion

In this cross-sectional analysis, the overall HEV seroprevalence was 6.3%. Concerning healthy controls, the IgG anti-HEV rate was very similar to that previously reported in blood donors from others European countries such as Switzerland (3.2%) [35] or the north of France (3.2%) [36]. Kidney-transplant recipients presented an anti-HEV seroprevalence of 3.5%, similar to the rate of the healthy donors (3.7%). A study from France showed also similar seroprevalence rates in both blood donors and kidney transplantation [19]. In regards to liver transplantation, the anti-HEV seroprevalence was 9.5%, a rate higher than previously reported in the Netherlands (3.2%) using the Genelabs assay [18] or in Germany (4%) where the Abbot ELISA was used but is not currently commercialized [17].

Regarding developed countries, hepatitis E seems to be related to zoonotic transmission of genotype 3 or 4 from an animal reservoir; however, the complete routes of transmissions and predictive factors of the development of acute and chronic infection are not totally understood, which hinders its prevention in non-epidemic settings [17], [18], [19], [26]. Moreover, HEV infection has been observed frequently affecting people with a suppressed immune system, even occasionally leading to persistent infection. Therefore, it is essential to know the burden of infection especially in populations with predisposition to infection. In this sense, seroprevalence studies assessing whether solid organ-transplant recipients are at a higher risk of HEV infection are scarce and the results discordant.

The underlying mechanisms causing differences between liver and kidney- transplant are not clear, however, recently, it has been also reported that KT patients are at lesser risk for HEV infection compared with liver-transplant [19]. It should be noted that all liver recipients underwent transplantation at end-stage liver disease, so the advanced degree of liver fibrosis could be a predisposing factor for HEV infection or, another likely explanation, would be that hepatitis E may play a role in the development of liver cirrhosis. Futhermore, we should underline that important differences have been described between the anti-HEV IgM commercial assays in immunocompromised patients, specifically solid-organ recipients (11% with Adaltis vs 31.3% with Wantai), so the same phenomena could be expected regarding anti-HEV IgG [37].

The most outstanding finding of our study is the high HEV seroprevalence in patients with liver cirrhosis (17.5%), particularly those who developed cirrhosis after transplantation (32.1%). This observation is also documented in HIV-infected patients, in which anti-HEV was 22.7% similar to the 22.4% rate in non-HIV patients with liver cirrhosis.

In agreement with our data, a report from India, a hyper-endemic area for HEV, also found an association between liver cirrhosis and HEV infection [38]. These data suggest that cirrhotic individuals could have a high risk of acquiring HEV infection, which is important since rapid deterioration of liver function and a high mortality rate have been reported in patients developing acute-on-chronic liver failure after acute hepatitis E infection [2], [27], [39]. An explanation for this finding could be the immune dysfunction observed in cirrhotic patients, who present decreased innate immune system activity with a reduction in natural killer cell activity [40]. Also in this line, innate immunity was found to be suppressed in advanced stages of liver fibrosis in an experimental mouse model of cirrhosis [41]. Moreover, HEV by itself can contribute to a downregulation of immune activity. In this sense, it has been reported that the protein encoded by HEV open reading frame (ORF) 3 gene might reduce the host inflammatory response, even attenuating the acute phase reaction, further creating an environment favourable for viral replication [42]. In fact, secretion of immunosuppressive α1-microglobulin was increased in HEV ORF3-protein expressing cells potentially resulting in a protection of of virus-infected cells [43]. The additive effect of both factors, innate-immunity suppression in advanced liver fibrosis and the direct immunosuppresive effect of HEV, could explain the higher HEV infection susceptibility of LC patients in relation to other groups.

Alternatively, HEV, which can evolve to chronicity in immunosuppressed patients, could be implicated in the pathogenesis of cirrhosis in this population, in whom a high percentage of patients are infected with HCV or HBV. However, anti-HEV seroprevalence was lower in patients coinfected with HCV than from the rest of liver disease aetiologies (6.4 vs 9.1%, p 0.1). Futhermore, in the multivariant analysis, hepatitis C infection was a protective factor for the presence of anti-HEV IgG. An explanation for this fact could be that treatment for chronic hepatitis C includes ribavirin, a therapy also effective against HEV [2], [17], [24], [39], [44], [45], [46].

Concerning HIV-infected patients, this patient cohort was previously analysed [30] but not compared with other inmunosupressed patients, this comparison has been performed in the present study. In this sense, hepatitis E seroprevalence in the HIV cohort was statistically higher than non-HIV population (9.2% vs 5.2%, p 0.03), a rate that increased when patients with CLD and particularly liver cirrhosis were analysed separately, being the IgG anti-HEV rate 10% and 22.7% respectively. In the literature, there are discrepancies with regards to the real seroprevalence of HEV in the HIV positive population and the possibility of a higher predisposition in this group to hepatitis E infection. On the one hand, some reports have shown higher IgG anti-HEV rate in HIV-infected [21], [47], [48], [49], in contrast to general, population. Moreover, Mateos-Lindemann ML *et al* has recently published an IgG anti-HEV rate of 10.4% in a cohort of HIV-infected patients from Spain [21], a rate higher than the previously reported 2.8% of the general population from that area of Spain [50]. On the other hand, reports from England and France, have shown a similar or even lower seroprevalence of HEV infection in HIV-infected patients [22], [51]. The reason for a possible higher anti-HEV seroprevalence in HIV-infected patients remains unknown, though this fact could be related to blood transmission of both infections. What is more, some cases of HEV infection transmitted by blood products have been reported worldwide [5], [6], [7], [8], [9], [10], [11], and also use of intravenous drugs has been associated to a higher IgG anti-HEV rate than in the general population [35].

A limitation of our study is the anti-HEV IgG test used. In recent years, the assays for the determination of anti-HEV have improved, and some studies have revealed that a Chinese assay, Wantai test, which is not commercialized in Europe, could be more sensitive [52], [53]. However, the aim of our study was not establishment of absolute prevalence, but the search for risk factors associated with HEV infection, and for this reason we test in the same manner a large cohort of immunocompromised patients, probably the most extensive to date.

In conclusion, the main insight of our study is the high HEV seroprevalence observed in liver-transplant patients and the strong association between anti-HEV with liver cirrhosis. These results suggest that HEV should be considered in the differential diagnosis of otherwise unexplained hepatitis and HEV screening should be implemented prior to liver transplantation.
